# Myosin1D is an evolutionarily conserved regulator of animal left–right asymmetry

**DOI:** 10.1038/s41467-018-04284-8

**Published:** 2018-05-16

**Authors:** Thomas Juan, Charles Géminard, Jean-Baptiste Coutelis, Delphine Cerezo, Sophie Polès, Stéphane Noselli, Maximilian Fürthauer

**Affiliations:** Université Côte d’Azur, CNRS, Inserm, Institut de Biologie Valrose, Nice, F-06108 France

## Abstract

The establishment of left–right (LR) asymmetry is fundamental to animal development, but the identification of a unifying mechanism establishing laterality across different phyla has remained elusive. A cilia-driven, directional fluid flow is important for symmetry breaking in numerous vertebrates, including zebrafish. Alternatively, LR asymmetry can be established independently of cilia, notably through the intrinsic chirality of the acto-myosin cytoskeleton. Here, we show that Myosin1D (Myo1D), a previously identified regulator of *Drosophila* LR asymmetry, is essential for the formation and function of the zebrafish LR organizer (LRO), Kupffer’s vesicle (KV). Myo1D controls the orientation of LRO cilia and interacts functionally with the planar cell polarity (PCP) pathway component VanGogh-like2 (Vangl2), to shape a productive LRO flow. Our findings identify Myo1D as an evolutionarily conserved regulator of animal LR asymmetry, and show that functional interactions between Myo1D and PCP are central to the establishment of animal LR asymmetry.

## Abstract (web summary)

Left-right (LR) axis specification is essential for embryonic patterning but a unifying mechanism across organisms has not been identified. Here, the authors show that Myosin1D, known to regulate *Drosophila* LR asymmetry, controls zebrafish LR Organizer function, and is therefore a conserved regulator of animal laterality.

## Introduction

The molecular pathways governing antero-posterior and dorsoventral patterning have been extensively conserved throughout evolution. In contrast, numerous studies have revealed a striking diversity in the strategies establishing laterality both within and across phyla, making the identification of a unifying mechanism elusive^1–4^. Processes that have been involved in symmetry breaking range from cilia-driven fluid flows^5–7^ and localized ion flows^8–10^ to cellular rearrangements dependent on cytoskeletal polarity^11–14^. Body laterality can even be subject to distinct regulations in a single organism^15,16^. This apparent diversity raises the question whether a unifying mechanism underlying the establishment of LR asymmetry still remains to be identified?

An attractive hypothesis is that the chirality of actin filaments may provide a template for LR asymmetry^1,17^. Accordingly, actin-binding proteins govern molecular^18^ and cellular chirality^19^ and actin-dependent processes regulate invertebrate and vertebrate laterality^1,15,20–23^. Studies in *Drosophila* have identified the actin-based molecular motor protein Myosin1D (Myo1D, a.k.a. Myosin31DF) as an essential regulator of LR asymmetry^20,21^. LR asymmetry in *Drosophila* governs the dextral rotation of male genitalia, as well as the looping of testis, midgut and hindgut. *myo1D* mutants show a LR inversion of all lateralized organs, identifying Myo1D as a key regulator of dextral development^24^. In the adult hindgut, functional interactions of Myo1D with planar cell polarity (PCP) pathway components govern cellular chirality and promote asymmetric gut looping^13^. Here we study the contribution of Myo1D to vertebrate laterality, by analyzing its function in zebrafish.

The zebrafish LR organizer (LRO), Kupffer’s vesicle (KV), is a transient vesicular organ that is decorated on its inside with motile cilia whose beating creates a counter-clockwise fluid flow essential for LR asymmetry^6^. This directional ciliary flow has been proposed to promote symmetry breaking through the local activation of mechanosensory Ca^2+^ channels such as PKD2 or the lateralized transport of signaling molecules in the extracellular space^8,25,26^. While the mechanism through which the flow contributes to chiral morphogenesis remains debated, it is firmly established that a directional LRO flow is essential for zebrafish LR asymmetry^6,25,26^. The generation of a functional LRO flow requires both polarized KV cell shape remodeling^14,27^, as well as a PCP-dependent control of the spatial orientation of flow-generating motile cilia^28^.

In the present study, we provide genetic evidence that *myo1D* is essential for the establishment of zebrafish LR asymmetry. Myo1D controls the morphogenesis and function of the zebrafish LRO by interacting functionally with the PCP pathway component VanGogh-like2 (Vangl2). KV cilia orientation is controlled by opposing activities of Myo1D and Vangl2, the balance of which is essential for the establishment of a directional KV flow and subsequent chiral morphogenesis. Taken together, our findings identify Myo1D as a common regulator of LR asymmetry whose function is conserved in both vertebrate and invertebrate organisms.

## Results

### Myo1D is essential for zebrafish left–right (LR) asymmetry

**Fig. 1:**
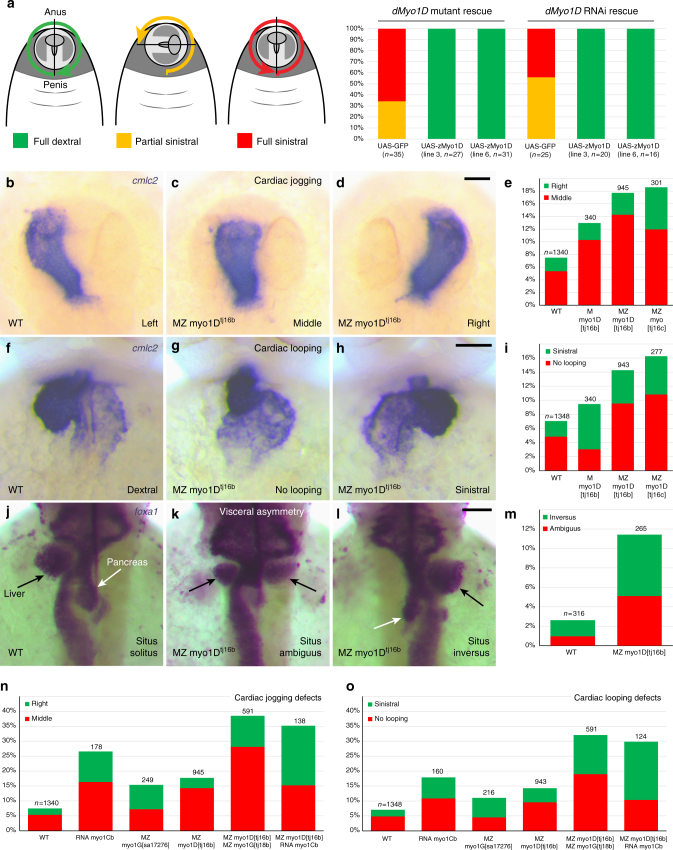
*myo1D* controls zebrafish left–right asymmetry. **a** Transgene-mediated expression of zebrafish *myo1D (zmyo1D)* restores the laterality of genitalia rotation in *Drosophila myo1D (dmyo1D)* mutant or *dmyo1D* RNAi flies. Lines 3 and 6 are two independent transgenic insertions. **b**–**e** Zebrafish MZ *myo1D* mutants present defects in leftward cardiac jogging. **b**–**d** Dorsal views of the *cmlc2*-expressing heart primordium at 26 hpf, anterior up. **e** Quantification of cardiac jogging defects in maternal (M) and maternal zygotic (MZ) *myo1D*^*tj16b*^ or *myo1D*^*tj16c*^ mutants. **f**–**i** Cardiac looping is affected in MZ *myo1D* mutants. **f**–**h** Frontal views of the *cmlc2*-expressing heart at 48 hpf, dorsal up. **i** Quantification of cardiac looping defects in M *myo1D*^*tj16b*^ and MZ *myo1D*^*tj16b*^ or MZ *myo1D*^*tj16c*^ mutants. **j**–**m** MZ *myo1D* mutants present defects in the leftward looping of the gut and the asymmetric development of the liver (black arrows) and pancreas (white arrows). **j**–**l** Dorsal views of *foxa1*-expressing visceral organs at 48 hpf, anterior up. **n**, **o** Overexpression of zebrafish *myo1Cb* RNA impairs cardiac jogging (**n**) and looping (**o**). MZ *myo1G* single mutants present laterality defects (**n**, **o**). MZ *myo1D*; MZ *myo1G* double mutants or MZ *myo1D* mutants injected with *myo1Cb* RNA present an increased frequency of defects compared to MZ *myo1D* mutants (**n**, **o**). Scale bars: 30 µm

We first determined whether zebrafish and *Drosophila* Myo1D have conserved activities by testing the ability of fish *myo1D* to rescue the laterality defects of fly *myo1D* mutants. The two orthologous proteins show 69% sequence similarity. Expressing zebrafish *myo1D* in *Drosophila* fully restored the chirality of genitalia rotation (Fig. 1a), a prominent LR marker in *Drosophila*^20,21^. These data indicate that zebrafish Myo1D is capable of mediating a conserved LR symmetry-breaking activity.

To study zebrafish *myo1D* function, we generated frameshift mutations that disrupt the P-loop required for Myo1D motor activity^29^ and delete the actin- and cargo-binding domains (Supplementary Fig. 1a). Homozygous mutants lacking zygotic *myo1D* function develop normally and give rise to fertile adults (Supplementary Fig. 1b). In situ hybridization reveals that, in addition to their zygotic expression, *myo1D* transcripts are maternally provided (Supplementary Fig. 1c–e). We therefore used homozygous mutant females to generate embryos lacking either only maternal (M) or both maternal and zygotic (MZ) *myo1D* activities. M and MZ *myo1D* mutants display LR asymmetry defects at the level of the heart (Fig. 1b–i), viscera (Fig. 1j–m), and brain (Supplementary Fig. 2a–f). Similar phenotypes were observed using two different mutant alleles (Fig. 1e, i). While LR defects of MZ *myo1D* mutant animals could be partially rescued through the injection of an RNA encoding wild-type (WT) Myo1D protein, no rescuing activity was observed using *myo1D* mutant RNA (Supplementary Fig. 2g–k). Similar defects are moreover generated when a morpholino that blocks the translation of both maternally deposited and zygotically transcribed *myo1D* RNA is injected in WT embryos (Supplementary Fig. 3). Altogether, our experiments identify Myo1D as a conserved regulator of laterality in both vertebrates and invertebrates.

In *Drosophila*, Myosin1C *(*Myo1C, a.k.a. Myo61F*)* acts as an antagonist of the essential dextral regulator Myo1D^30^, with *myo1C* overexpression phenocopying *myo1D* loss of function. However, *Drosophila myo1C* is itself dispensable for LR asymmetry^30^. The zebrafish genome encodes two *myo1C* homologues, *myosin1Ca* and *myosin1Cb* (*myo1Ca* & *myo1Cb*). Like in *Drosophila*, zygotic *myo1Ca* mutants and MZ *myo1Cb* mutants do not display LR defects (not shown). Conversely, Myo1Cb overexpression using mRNA microinjection causes cardiac laterality defects (Fig. 1n, o), thus mimicking *myo1D* loss of function, as observed in *Drosophila*^30^.

Overexpression of zebrafish *myo1Cb* enhances MZ *myo1D* mutant LR defects (Fig. 1n, o), raising the question whether additional Myo1D-like activities might contribute to the regulation of LR asymmetry. Genome analysis shows that zebrafish Myosin1G (Myo1G) is closely related to Myo1D (79% similar amino acids). To invalidate *myo1G*, we used two mutant alleles that introduce premature stop codons at different positions of the open reading frame (see Methods). Zygotic *myo1G* single mutants developed normally into fertile adults, but MZ *myo1G* mutant embryos displayed cardiac laterality defects (Fig. 1n, o). MZ *myo1D*; MZ *myo1G* double mutants were created through Crispr/Cas9 mutagenesis of the *myo1G* locus in a *myo1D* mutant background (see Methods). MZ *myo1D*; MZ *myo1G* double mutants presented an increased frequency of LR defects compared to *myo1D* single mutants, similar to the one observed upon *myo1Cb* overexpression (Fig. 1n, o). Our findings provide evidence that Myo1D-like agonists (Myo1D & G) and their antagonist Myo1Cb form an evolutionarily conserved pathway for the control of LR asymmetry.

### Myo1D is required for LRO function

**Fig. 2:**
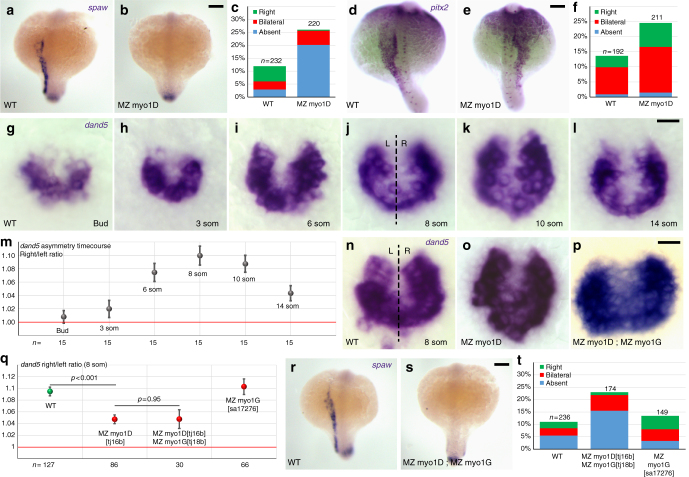
myo1D is required for left–right Organizer function. **a**–**f** MZ *myo1D* mutants display an impaired expression of *southpaw (spaw)* and *pitx2* in the left lateral plate mesoderm. **c**, **f** Quantifications of *spaw* (**c**) and *pitx2* (**f**) expression. For *pitx2*, the most marked difference between WT and MZ *myo1D* mutants is observed in the category with right-sided expression (**e**). **g**–**m** Temporal evolution of the asymmetry of *dand5* expression between the end of gastrulation (Bud) and the 14-somite stage. **m** Quantifications of the relative abundance of *dand5* transcripts on the left (L) and right (R) side of WT ABTÜ embryos reveal a peak of LR asymmetry at the eight-somite stage. **n**–**q** Eight-somite stage asymmetry of *dand5* asymmetry is reduced in MZ *myo1D* mutants. No further reduction of asymmetry is observed in MZ *myo1D*; MZ *myo1G* double mutants. For values of *dand5* asymmetry for individual display items, see Methods. **r**–**t** Asymmetric *spaw* expression is altered in MZ *myo1D*; MZ *myo1G* double mutants. The frequency of defects is comparable to MZ *myo1D* single mutants (**c**). **a**, **b**, **d**, **e**, **r**, **s** are dorsal views of 18-somite stage embryos, anterior up. **g**–**l** and **n**–**p** are dorsal views of the LRO, anterior up. Error bars in **m** and **q** indicate SEM. Scale bars: 50 µm in **a**, **b**, **d**, **e**, **r**, **s**; 20 µm in **g**–**l** and **n**–**p**

The observation that MZ *myo1D* mutants present laterality defects at the level of the heart, viscera, and brain (Fig. 1b–m, Supplementary Fig. 2a–f), suggests that *myo1D* may control the formation and/or function of the central zebrafish LRO, KV^6,31^. The ciliary LRO flow induces the expression of both the nodal ligand *southpaw (spaw)* and the transcription factor *pitx2* in the left lateral plate mesodem^32,33^. Consistent with a potential role of Myo1D at the level of the LRO, the asymmetric expression of these two genes is disrupted in MZ *myo1D* mutants (Fig. 2a–f). To further test Myo1D function in the LRO, we looked at the asymmetric expression of the TGFβ antagonist *charon/dand5*, which is the earliest known transcriptional response to the KV flow^25,34^. While weak symmetric *dand5* expression is initiated prior to the establishment of a functional KV, *dand5* asymmetry arises as the LRO matures, peaks by the eight-somite stage and decreases again during later development (Fig. 2g–m). In accordance with a dysfunction of the LRO, *dand5* asymmetry is reduced in MZ *myo1D* mutants (Fig. 2n, o, q). These data demonstrate that Myo1D is required prior to the first morphological manifestations of LR asymmetry for the LRO-dependent establishment of asymmetric gene expressions.

### Myo1D controls the ciliary LRO flow

**Fig. 3:**
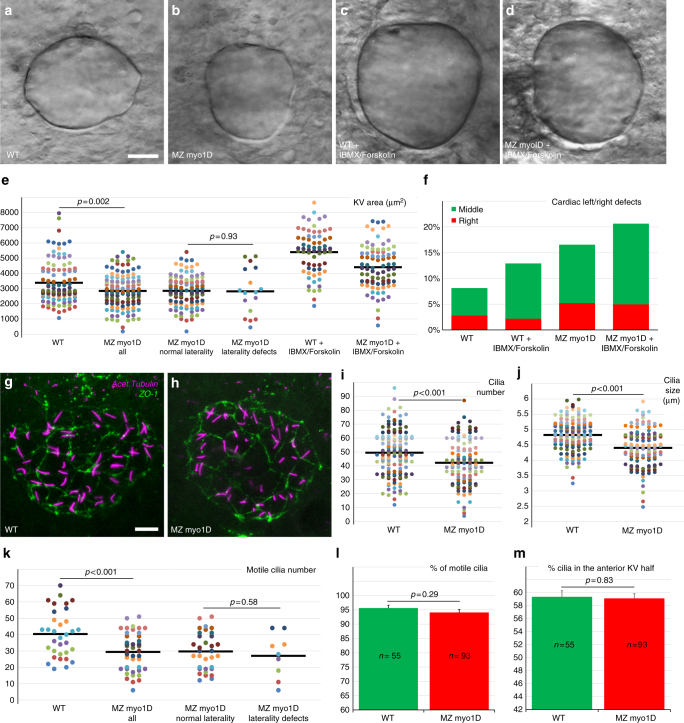
*myo1D* controls the morphogenesis of the zebrafish left–right Organizer. **a**–**e** Compared to WT (*n* = 104), MZ *myo1D* mutants (*n* = 118) present a reduced KV size. Treatment with IBMX and Forskolin promotes KV lumen inflation and increases organ size in WT (*n* = 66) and MZ *myo1D* mutants (*n* = 85). **e** Dot plots of KV equatorial surface area in individual embryos. KV size is similar in embryos with normal or defective laterality. **f** IBMX/Forskolin treatment restores KV size (**d**, **e**), but not laterality in MZ *myo1D* mutants. **g**–**j** Cilia number and size are reduced in MZ *myo1D* mutants. **g**, **h** Projection of images from confocal stacks used to quantify number and length of cilia (acetylated tubulin, magenta) in the KV (ZO-1 positive cells, green). **i**, **j** Number and average length of cilia in individual embryos are reduced in MZ *myo1D* (*n* = 117 embryos/4939 cilia) compared to WT (*n* = 141/6967). **k**–**m** Confocal imaging of Arl13b-GFP labeled cilia in living embryos was used to analyze the number (**k**), motility (**l**) and positioning (**m**) of KV cilia. **k** Dot plot representing motile cilia number in individual embryos. MZ *myo1D* (*n* = 42) mutants display lower motile cilia numbers compared to WT (*n* = 33). Motile cilia numbers are however similar in MZ *myo1D* mutant embryos with normal or defective laterality. Total cilia numbers for this experiment are displayed in Supplementary Fig. 5. **l** *myo1D* loss of function does not impair ciliary motility. **m** WT and MZ *myo1D* mutant animals display a similar enrichment of cilia in the anterior KV half. **a**–**d**, **g**, **h** are dorsal views of the KV, anterior up. All data collected at the eight-somites stage. Horizontal bars in **e**, **i**, **j**, **k** represent mean values. Error bars in **l**, **m** represent SEM. Scale bars: 20 µm in **a**–**d**; 10 µm in **g**–**h**

To further dissect the role of *myo1D* in zebrafish LR asymmetry, we analyzed its contribution to the formation and function of the KV. While *myo1D* is dispensable for the migration and coalescence of *sox17*-positive KV precursor cells (Supplementary Fig. 4), we found that the average KV size and the total number of cilia are reduced in MZ *myo1D* mutants (Fig. 3a, b, e, g–i). LRO function requires a minimal organ size and number of motile cilia^25,35^. We monitored KV size, total, and motile cilia number together with laterality in individual embryos. No correlation could be observed between organ size, cilia numbers, and the appearance of laterality defects (Fig. 3e, k, Supplementary Fig. 5). KV lumen inflation is promoted through the activation of the cystic fibrosis transmembrane conductance regulator (CFTR) channel^36^. Accordingly, treatment with IBMX and Forskolin, which activate CFTR through an elevation of cAMP levels and PKA activity, rescues KV size but not laterality defects in MZ *myo1D* mutants (Fig. 3c–f). While the loss of *myo1D* causes a modest reduction of cilia size (Fig. 3g, h, j), it has no incidence on the percentage of motile cilia (Fig. 3l). Previous studies have revealed that the majority of cilia are positioned in the anterior KV half^37^. This antero-posterior asymmetry is unaffected in MZ *myo1D* (Fig. 3m).

**Fig. 4:**
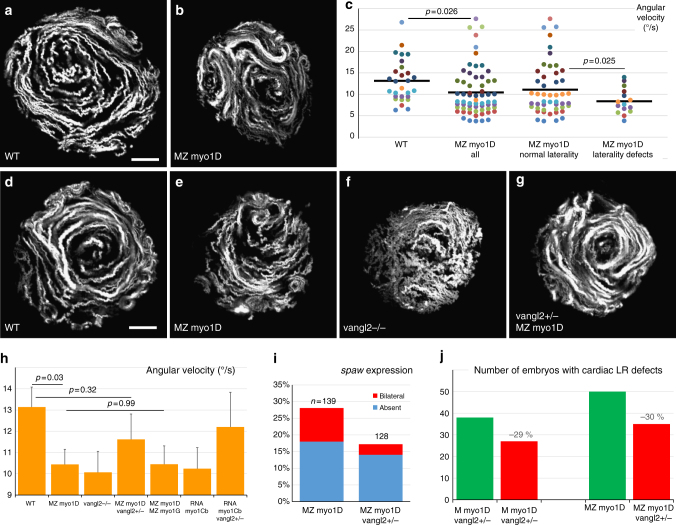
*myo1D* is required for the establishment of zebrafish left–right organizer flow. **a**–**c** KV flow is altered in MZ *myo1D* mutants. Temporal projections of the trajectories of fluorescent microspheres in WT (**a**, *n* = 28) and MZ *myo1D* mutants (**b**, *n* = 63). **c** MZ *myo1D* embryos display lower mean angular KV flow velocities than WT. MZ *myo1D* mutants with defective cardiac jogging show lower velocities than their sibling with normal laterality. **d**–**g** MZ *myo1D* (**e**) and *vangl2* (**f**, *n* = 36) mutants present an altered LRO flow compared to WT (**d**) but flow pattern is restored in MZ *myo1D* *;* *vangl2+/−* (**g**, *n* = 25). **h** MZ *myo1D* mutants display a reduced KV flow velocity compared to WT, as do *vangl2−*/− embryos (*p* = 0.03). Flow in MZ *myo1D* *;* *vangl2+/−* is however similar to WT. Flow velocity in MZ *myo1D*; MZ *myo1G* double mutants is reduced compared to WT (*n* = 29, *p* = 0.04) but similar to MZ *myo1D* single mutants. Flow velocity in WT embryos injected with *myo1Cb* RNA is reduced compared to WT controls (*n* = 37, *p* = 0.04). Removal of one copy of *vangl2* makes flow velocity again similar to WT (*n* = 19, *p* = 0.62). **i**, **j** The removal of one copy of *vangl2* reduces defects in asymmetric spaw expression (**i**) and cardiac morphogenesis (**j**) in MZ *myo1D* (see Methods). **a**, **b**, **d**–**g** are dorsal views of eight-somites stage KVs, anterior up. Display items and quantifications in **a**–**h** are derived from the same dataset. Horizontal bars in **c** represent mean values. Error bars in **h** indicate SEM. Scale bars: 20 µm in **a**, **b**, **d**–**g**

To visualize the LRO flow, we tracked fluorescent microspheres in the KV lumen. While WT controls mostly displayed circular trajectories (Fig. 4a), MZ *myo1D* mutants often presented aberrant flow patterns (Fig. 4b). To quantitatively assess the LRO flow, we determined its mean angular velocity as a measure of the effective circular flow strength (see Methods). MZ *myo1D* mutants display a decrease in angular flow velocity (Fig. 4c). Importantly, LR asymmetry defects in MZ *myo1D* mutants correlate with pronounced reductions in flow velocity (Fig. 4c). In contrast, treatment of control WT embryos with Ouabain to pharmacologically reduce KV size leads to an increase in flow velocity with no associated LR defects (Supplementary Fig. 6). These data show that *myo1D* is required for the establishment of a functional LRO flow, independently of KV size.

### Myo1D interacts with Vangl2 to control LR asymmetry

How does *myo1D* control KV flow? Embryos with no, or immotile, cilia lack a detectable flow^6,25,38^ and show highly penetrant LR asymmetry phenotypes (Supplementary Fig. 7). In MZ *myo1D* mutants, cilia are motile but the LRO flow is erratic, a phenotype reminiscent of mutants for the PCP pathway component *vangl2*, which displays less penetrant LR defects (Fig. 4d–f, Supplementary Fig. 7)^28,39^. We found that both MZ *myo1D* and *vangl2* mutants display a reduction in overall angular velocity and a disruption of the KV flow pattern (Fig. 4h, Supplementary Fig. 8a). While *myo1D* and *vangl2* mutants have similar flow phenotypes, the removal of one copy of *vangl2* partially rescues the KV flow defects of MZ *myo1D* embryos (Fig. 4g, h, Supplementary Fig. 8a), suggesting that the two genes affect the LRO in opposite ways. In accordance with the restoration of a functional KV flow, the removal of one copy of *vangl2* decreases the penetrance of LR asymmetry defects in *myo1D*-deficient animals (Fig. 4i, j).

A reduction of overall KV angular flow velocity and a disruption of circular flow geometry are also observed in MZ *myo1D*; MZ *Myo1G* double mutants or embryos injected with *myo1Cb* antagonist RNA (Fig. 4h, Supplementary Fig.8a–d), confirming the importance of the Myosin1 pathway for the control of LRO flow. Removal of one copy of *vangl2* allows to partially restore the KV flow defects that are elicited by *myoICb* overexpression (Fig. 4h, Supplementary Fig. 8a, d, e), further supporting the existence of functionally relevant Myo1/PCP interactions. Our velocity measurements do not allow to explain the enhanced LR asymmetry defects of MZ *myo1D*; MZ *Myo1G* double mutants or *myo1Cb-*injected animals compared to MZ *myo1D* single mutants (Fig. 1n, o). This may be due to the fact that these measurements do not encompass the full complexity of the observed flow phenotypes. Alternatively, zebrafish Myosin1 proteins could act at different steps of the chiral morphogenesis program, in addition to their importance for LRO flow. In accordance with this hypothesis, similar defects are observed in MZ *myo1D* single and MZ *myo1D* *;* MZ *myo1G* double mutants for the LRO-dependent expression of *dand5* and *spaw* (Fig. 2a–c, n–t). This situation is consistent with the known role of *Drosophila myo1D*, which controls the morphogenesis of different organs through distinct cellular mechanisms^13,21,30^.

**Fig. 5:**
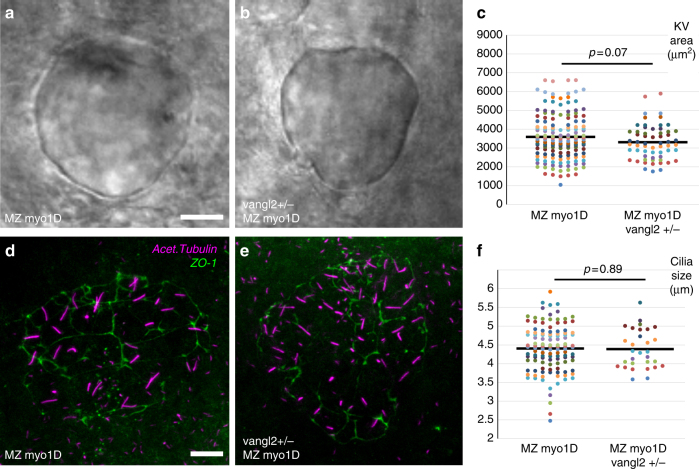
KV area and cilia size are similar in MZ *myo1D* and MZ *myo1D vangl2+/−*. **a**–**c** MZ *myo1D* (*n* = 156) and MZ *myo1D ; vangl2+/−* (*n* = 60) animals have similar KV sizes. **d**–**e** Projection of images from confocal stacks used to quantify the length of cilia (acetylated tubulin, magenta) in the KV (ZO-1 positive cells, green) of MZ *myo1D* mutants (**d**), or MZ *myo1D ; vangl2+/−* embryos (**e**). **f** MZ *myo1D* (*n* = 117 embryos/4939 cilia) and MZ *myo1D ; vangl2+/−* (*n* = 30/1653) embryos present a similar cilia size. The MZ *myo1D* mutant dataset displayed in **f** is the same that is also displayed in Fig. 3j. **a**, **b**, **d**, **e** are dorsal views of eight-somites stage KVs, anterior up. Scale bars: 20 µm in **a**, **b**; 10 µm in **d**, **e**

How do *myo1D* and *vangl2* exert opposite effects on LRO morphogenesis and function? In accordance with our previous conclusion that the reduction of KV size is not the primary cause of MZ *myo1D* LR asymmetry defects (Fig. 3e), removal of one copy of *vangl2* has no significant effect on the KV size of MZ *myo1D* mutant animals (Fig. 5a–c). Similarly, reducing *vangl2* gene dosage does not restore the reduction of cilia length that is observed in MZ *myo1D* mutants (Figs. 3j and 5d–f).

**Fig. 6:**
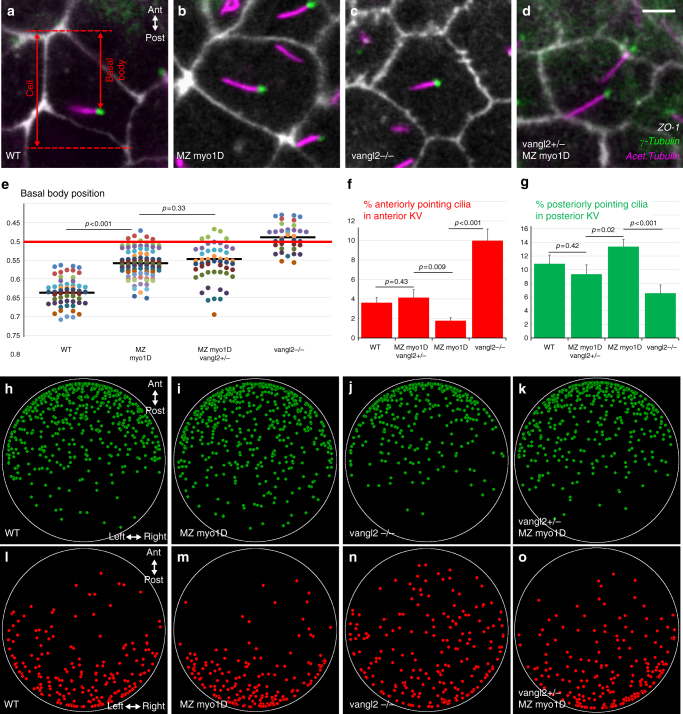
*myo1D* interacts with *vangl2* to control cilia orientation in the zebrafish left–right Organizer. **a**–**e** *myo1D* and *vangl2* are required for basal body positioning. **a**–**d** High magnification views of KV cells (ZO-1) with cilia (acetylated tubulin) and basal bodies (γ-tubulin). **e** Dot plot representing average antero-posterior basal body position in individual embryos (see Methods). Compared to WT (**a**, *n* = 40 embryos/744 basal bodies), basal bodies are displaced anteriorly in MZ *myo1D* (**b**, *n* = 74/1521) or *vangl2* (**c**, *n* = 36/526) mutants. Basal bodies are similarly affected in MZ *myo1D* and MZ *myo1D ; vangl2+/*− (**d**, *n* = 37/826). **f**, **g** MZ *myo1D* (*n* = 93 embryos/3172 cilia) and *vangl2* (*n* = 50/1545) mutations have opposing effects on cilia orientation. Cilia orientation in MZ *myo1D ; vangl2+/*− (*n* = 35/1225) is similar to WT (*n* = 55/2275). **h**–**o** two-dimensional dot plots indicating the position of KV cilia pointing to the anterior (red) or posterior (green) in the KV of embryos of different genotypes. Posteriorly pointing cilia invade the posterior KV in MZ *myo1D* (**i**), while anterior pointing cilia invaded the anterior KV in *vangl2−/−* (**n**). In MZ *myo1D ; vangl2+/*− (**k**, **o**), cilia distribution is again more similar to WT (see Supplementary Fig. 9 for details). All data collected at eight-somites stage, anterior up in **a**–**d**, **h**–**o**. Horizontal bars in **e** represent mean values. Error bars in **f**, **g** indicate SEM. Scale bar: 5 µm in **a**–**d**

PCP signaling controls the posterior positioning of the ciliary basal body and the orientation of the ciliary rotation cone to promote LRO flow^11,28,40,41^. Rat *myo1D* controls cilia orientation in multiciliated epithelia^42^. In this context, *myo1D* governs both the positioning of ciliary basal bodies (reflecting translational PCP) and the orientation of the ciliary rotation cone (rotational PCP). The inactivation of either *myo1D* or *vangl2* causes an anterior displacement of the basal body in the zebrafish LRO (Fig. 6a–e). The observation that basal body positioning is similar in MZ *myo1D* and MZ *myo1D ;* *vangl2+/−* animals (Fig. 6b, d, e) suggests however that the two factors do not interact in the control of translational PCP. To determine the effect of *myo1D* and *vangl2* on rotational PCP, we imaged cilia in live embryos and found that both genes affect cilia orientation: while *vangl2* mutants present an excess of anteriorly pointing cilia in the anterior KV, the loss of *myo1D* increases the number of posteriorly pointing cilia in the posterior organ half (Fig. 6f–o, Supplementary Fig. 9). Importantly, the removal of one copy of *vangl2* significantly alters the cilia orientation of MZ *myo1D* mutants, so that MZ *myo1D* ; *vangl2+/−* animals are similar to WT controls (Fig. 6f, g). These findings suggest that *myo1D* and *vangl2* interact in the context of the control of ciliary rotational PCP, but do so in opposite ways, consistent with our genetic interaction data (Fig. 4d–j). Taken together, our data suggest that *myo1D*/PCP interactions are required to govern KV cilia orientation and promote the establishment of a functional directional flow in the zebrafish LRO.

## Discussion

The existence of an evolutionarily conserved mechanism for the establishment of animal LR asymmetry has long been subject to debate^1–4^. Here we identify Myo1D, an unconventional non-muscle Myosin that was initially identified for its essential function in *Drosophila* LR asymmetry^20,21^, as an evolutionarily conserved regulator of animal LR asymmetry. Our genetic analysis reveals that laterality is correctly established in zebrafish embryos that lack zygotic *myo1D* function, similar to zygotic rat *myo1D* mutants^42^. In contrast, laterality defects are observed in zebrafish MZ *myo1D* mutants (Fig. 1b–i). While a single copy of *myo1D* is present in flies, the zebrafish genome encodes *myo1D* and the closely related *myo1G*. MZ *myo1D;* MZ *myo1G* double mutants present an increased penetrance of LR asymmetry defects compared to MZ *myo1D* single mutants, revealing an essential function of *myo1G* in the establishment of vertebrate laterality (Fig. 1n, o). As in *Drosophila*, chiral morphogenesis is disrupted upon overexpression of the Myo1D-antagonist Myo1Cb (Fig. 1n, o). These findings suggest that Myo1D-like antagonists and Myo1C antagonists represent an evolutionarily conserved toolkit for the establishment of animal laterality.

MZ *myo1D* mutants present defects in cardiac, visceral, and brain laterality (Fig. 1b–m, Supplementary Fig. 2a–f), suggesting that *myo1D* function may be required at the level of the KV, which acts as a central zebrafish LRO. Accordingly, the inactivation of *myo1D* disrupts LRO-dependent asymmetric gene expressions at the level of the KV (*dand5*) and the left lateral plate mesoderm (*pitx2*) (Fig. 2). Through a quantitative analysis of KV architecture and LRO fluid flow in MZ *myo1D* mutants, we identify the failure to establish a functional LRO flow as the primary cause of MZ *myo1D* mutant laterality defects (Fig. 4a–c).

MZ *myo1D* mutants present an alteration of flow geometry and circularity that are similar to the ones resulting from the inactivation of the PCP pathway component *vangl2*^28,39^ (Fig. 4d–f), suggesting that these two factors control the same biological process. Accordingly, a lowering of *vangl2* gene dosage allows to partially rescue KV flow and embryonic laterality in MZ *myo1D* mutants (Fig. 4g–j). Studies in different vertebrate model organisms have revealed that PCP pathway activity governs the position and orientation of LRO cilia^11,28,40,41^. We provide evidence that, as in other species, basal bodies are located in the posterior half of zebrafish LRO cells. Inactivation of *myo1D* or *vangl2* results in an anterior displacement of the basal bodies (Fig. 6a–e). In contrast, *myo1D* and *vangl2* mutants present opposite defects in cilia orientation (Fig. 6f–o). The reduction in *vangl2* gene dosage that partially rescues KV flow and morphological laterality defects restores the rotational orientation, but not the basal body position, of LRO cilia (Fig. 6e–g). Translational and rotational PCP of KV cilia can therefore be uncoupled, and the sole restoration of rotational PCP is sufficient to achieve a significant rescue of LRO flow.

**Fig. 7:**
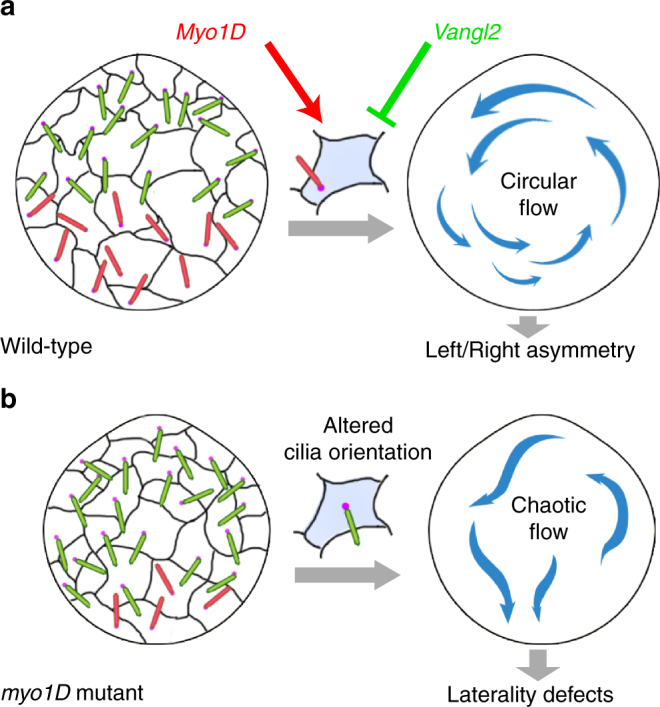
Model of Myo1D function in the zebrafish left–right organizer. **a** Schematic representation of a wild-type Kupffer’s vesicle (KV). Black lines indicate cell outlines and dots in magenta the basal bodies of KV cilia. Cilia pointing to the posterior are indicated in green, anteriorly pointing cilia in red. Under wild-type conditions Myo1D and Vangl2 exert opposing activities on cilia orientation, restricting thereby posteriorly pointing cilia to the anterior KV and anteriorly pointing cilia to the posterior KV. This control of cilia orientation allows the generation of a circular counter-clockwise fluid flow that promotes a proper establishment of left–right asymmetry at the level of different organs. **b** In *myo1D* mutant embryos, cilia orientation is altered so that posteriorly pointing cilia now invade the posterior KV. The KV flow loses its circular geometry and becomes chaotic, causing thereby the occurrence of subsequent morphological laterality defects at the level of the heart, viscera, and brain

Our observations suggest that Myo1D exerts an essential role in controlling the rotational orientation of zebrafish LRO cilia. Under WT conditions, the opposing activities of Myo1D and Vangl2 control cilia orientation to generate a productive LRO flow (Fig. 7a). The loss of either Myo1D or Vangl2 function disrupts this cellular tug of war, resulting in altered cilia orientations, chaotic LRO flow profiles, and ultimately LR asymmetry defects (Fig. 7b).

The molecular mechanism through which *myo1D* and *vangl2* interact remains to be established. A first possibility could be that *myo1D* functions as a general PCP pathway component. We do not favor this hypothesis. In addition to its function in the LRO, *vangl2* regulates the PCP-dependent positioning and orientation of floor plate cilia^28^. In contrast, *myo1D* has no effect on floor plate cilia (Supplementary Fig. 10a, b). Similarly, MZ *myo1D* mutants do not present defects in *vangl2*/PCP-dependent convergence-extension movements (Supplementary Fig. 11). These observations suggest that *myo1D* and *vangl2* interact specifically in the context of LR asymmetry, as observed in *Drosophila*^13^.

In the *Drosophila* adult hindgut, *myo1D* interacts differently with the global *fat/dachsous* and the core *vangl2* PCP pathways^13^. Myo1D is expressed in a transient LRO where it binds the global PCP component Dachsous to trigger Vangl2-dependent chiral morphogenesis in an adjacent tissue. *myo1D* and *vangl2* therefore interact genetically but not molecularly. In accordance with a similar scenario, Myo1D loss of function does not alter Vangl2 localization in the zebrafish LRO (Supplementary Fig. 10c, d).

A major challenge for future studies will be to determine how Myosin1 proteins interact with other components of the chiral morphogenesis program. It will notably be interesting to determine whether the residual KV flow of MZ *myo1D* mutant embryos is due to the activity of additional actin-dependent regulators of LR activity.

Taken together, our data show that Myo1D is essential for the formation and function of the zebrafish LRO, identifying a common regulator of LR asymmetry in both vertebrates and invertebrates. The functional interaction between zebrafish Myo1D and PCP controls the orientation of KV cilia that are required for the establishment of a directional, symmetry-breaking flow. Interestingly, recent work has shown a functional link between Myo1D and PCP pathways in *Drosophila*^13^ and in *Xenopus* (M. Blum, personal communication). We propose that the coupling between Myo1D and PCP may represent an evolutionarily conserved genetic framework to control animal LR asymmetry.

## Methods

### Fly strains and genetics

Flies were grown according to standard procedures. For inter-specific rescue experiments, the *UAS::zMyo1D* transgene (Zebrafish Myo1D ORF cloned into pUAST vector) was expressed either in *myo1D* depleted flies (*w*^*1118*^ *; ptc::GAL4, myo1D*^*k1*^ *; UAS::dmyo1D-RNAi*^21^) or in a *myo1D*^*k2 21*^ null mutant background (*da::GAL4* ; *myo1D*^*k2*^) (*da::GAL4*, Bloomington Drosophila Stock Center #55851). Two independent insertions of the *UAS::zMyo1D* transgene (lines 3 and 6) were used and showed similar results.

Rotation of the male genitalia was scored as previously described^43^. Briefly, male genital plates are scored according to the angle made between the dorsoventral and anus–penis axes when viewed from the posterior. Full dextral/clockwise rotation (+360°) corresponding to the WT situation; partial sinistral/counter-clockwise rotation (from –1 to –359°); full sinistral/counter-clockwise rotation (−360°).

### Zebrafish strains and embryo maintenance

Embryos were raised in 0.3X Danieau medium (17.4 mM NaCl, 0.21 mM KCl, 0.12 mM MgSO_4_, 0.18 mM Ca(NO3)_2_, 1.5 mM Hepes, pH 7.6) at 28.5 °C, and staged according to standard criteria^44^. If necessary, 1-phenyl-2-thiourea (Sigma) was added at 30 mg/l to prevent embryonic pigmentation.

### Crispr and Cas9 mutagenesis

Crispr/Cas9 mutagenesis of zebrafish *myo1D* (Ensembl gene ENSDARG00000036863) was performed in a WT TÜ background. gRNA design and in vitro transcription of Cas9 RNA were performed according to reported protocols^45^. gRNA was transcribed from a template oligo as previously described^46^. The sequence of the selected Crispr target site was 5′-GAGTGGAGCTGGAAAAACAG**AGG**-3′ (bold lettering indicates the PAM motif). The efficiency of Crispr/Cas9-induced mutagenesis was monitored at 24 hpf using a T7 endonuclease assay^45^ on a PCR amplicon comprising the Crispr target region (Forward primer: 5′-TCTTCACTGACACTGGTATG-3′, Reverse primer: 5′-CCATCACTGCAGCAGAAATGAGAG-3′). Adult F0 fish were outcrossed to AB WT fish and DNA extracted from F1 progeny. Mutations were identified through direct sequencing of the same PCR amplicon that was used in the T7 endonuclease assay.

To genetically invalidate zebrafish *myo1G* (Ensembl gene ENSDARG00000036104), we used two complementary experimental strategies. A first mutant allele (*myo1G*^*sa17276*^) was generated using a TILLING-based approach by the Zebrafish Mutation Project (http://www.sanger.ac.uk/resources/zebrafish/zmp/) and obtained through the Zebrafish International Resource Center. The sa17276 point mutation causes the appearance of a premature stop codon in place of amino acid 460, deleting therefore the entire C-terminal half of the protein including the actin- and cargo-binding domains.

To generate *myo1D ; myo1G* double mutants, a second mutant allele (*myo1G*^*tj18b*^) was created through Crispr/Cas9 mutagenesis of the *myo1G* locus (genomic target site 5′-GATGTCATTGAGGACTACAG**GGG**-3′) in a *myo1D* mutant background. A preassembled complex of purified Cas9 protein (NEB, 954 ng/µl) and *myo1G* gRNA (200 ng/µl) was injected into one cell stage embryos derived from an incross if *myo1D*^*tj16b/tj16c*^ *trans*-heterozygous parents and the cutting efficiency monitored using a T7 endonuclease assay on a *myo1G* PCR amplicon (Forward primer: 5′-GGAGAAGTAGTGGTGTCCGTTAAC-3′, Reverse primer: 5′-CTCACTTTTGGGCTAACAGCTC-3′). Injected F0 embryos were raised to adulthood, crossed to *myo1D*^*tj16b*^ homozygous fish, and DNA extracted from F1 progeny. Direct sequencing of the same PCR amplicon used in the T7 endonuclease assay lead to the identification of the *myo1G*^*tj18b*^ allele. The *myo1G*^*tj18b*^ mutation deletes a CA dinucleotide corresponding to positions 198/199 of the open reading frame and therefore causes the appearance of a premature stop codon in place of amino acid 66. Consequently, the Myo1G^tj18b^ mutant protein lacks all major functional domains, including the ATP-binding P-loop as well as actin- and cargo-binding regions.

### Fish strains and molecular genotyping

The following lines were used for this work: Tg(-5.0*sox17*:EGFP)zf99^47^, Tg(*cmlc2*:RFP)^48^, *vangl2*^m209^^49^, Tg(vangl2:Vangl2-GFP)^50^, and *dnaaf/lrrc50*^*tm317b 38*^.

WT and mutant alleles of *myo1D* were identified through allele-specific PCRs. The allele-specific forward primers 5′-TGGAGCTGGAAAAAGGCTCGT-3′ (*myo1D*^*tj16b*^) and 5′-GTGGAGCTGGAAAAAGGCTATAC-3′ (*myo1D*^*tj16c*^) were used together with the generic reverse primer 5′-CCATCACTGCAGCAGAAATGAGAG-3′ to genotype for the presence of different *myo1D* mutant alleles. PCR amplicon size was 133 bp for *myo1D*^*tj16b*^ and 145 bp for *myo1D*^*tj16c*^. The WT *myo1D* allele was amplified using the forward primer 5′-AGAGTGGAGCTGGAAAAACAGA-3′ and the reverse primer 5′-CCCATCCCTCGTGTGAAACTAAATCAC-3′ to yield a 339 bp amplicon.

The allele-specific reverse primers 5′-CCCTGCAATGAGACAGGCTTA-3′ (recognizing the *myo1G*^*sa17276*^ mutation) and 5′-CCCTGCAATGAGACAGGCGTC-3′ (recognizing the corresponding WT sequence) were used together with the generic forward primer 5′-AGGAGTACCAGAGAGAGGGAATC-3′ to detect the presence of the *myo1G*^*sa17276*^ mutant allele (215 bp amplicons). The allele-specific reverse primers 5′-TCTCATACAGTTCTCTTCCCCTAG-3′ (*myo1G*^*tj18b*^, 115 bp amplicon) and 5′- CTCATACAGTTCTCTTCCCCTGTAG-3′ (WT, 120 bp amplicon) were used together with the generic forward primer 5′-GAGAAGAGTCGTATCTACACCTTC-3′ to detect the presence of the *myo1G*^*tj18b*^ mutant allele.

PCR amplifications were performed with GoTaqG2 polymerase (Promega) at 1.5 mM MgCl_2_ using the following cycling conditions: Initial denaturation 2 min 95 °C; 10 cycles (30 s 95 °C, 30 s 65 °C first to 55 °C last cycle, 30 s 72 °C); 20 cycles (30 s 95 °C, 30 s 55 °C, 30 s 72 °C); final extension 5 min 72 °C.

To generate M zygotic *myo1D* mutants (MZ *myo1D*^*tj16b/tj16b*^ or MZ *myo1D*^*tj16c/tj16c*^), *myo1D* heterozygous fish were incrossed and the progeny raised to adulthood. PCR genotyping of these animals allowed identifying homozygous mutant, as well as homozygous WT fish which were then used to produce MZ *myo1D*^*tj16b/tj16b*^ or MZ *myo1D*^*tj16c/tj16c*^ mutant embryos, as well as WT *myo1D*^*+/+*^ control embryos. Unless mentioned otherwise, experiments were performed using the *myo1D*^*tj16b*^ allele. Similar procedures were used to generate MZ *myo1G*^*sa17276*^ single and MZ *myo1D*^*tj16b*^ ; MZ *myo1G*^*tj18b*^ double mutants.

For the analysis of *spaw* expression in MZ *myo1D* ; *vangl2+/−* animals presented in Fig. 4i, *myo1D*^*tj16b/tj16b*^ females were crossed to *myo1D*^*tj16b/tj16b*^ ; *vangl2+/−* males. Following in situ hybridization, a total of 267 animals were first scored for *spaw* expression and then genotyped for the presence of the *vangl2*^*m209*^ allele as previously described^51^. PCR was performed with forwards primers specific for either the WT (5′- GTGTGTCTGCCTGTGTCTTACT-3′) or the mutant (5′-GTGTGTCTGCCTGTGTCTTACA-3′) allele together with a generic reverse primer (5′- GATAAACTCCTCCCCCAGGT-3′) to amplify 250 bp amplicons.

For the genetic interaction experiment displayed in Fig. 4j, *myo1D*^*tj16b*^ homozygous mutant females were crossed with *myo1D*^*tj16b/+*^*; vangl2*^*m209/+*^ males. A total of 912 embryos derived from six independent crosses were scored and molecular genotyping performed for all embryos presenting cardiac jogging defects.

### Plasmid generation

The *myo1Cb* ORF was amplified from a mixed stage pool of cDNAs using the primers: 5′- **GATCCCATCGATTCGA**CGGAATCCGGGTCATGATG-3′ and 5′-**AGGCTCGAGAGGCCTT**GGTCACGACTCGTCCATC-3′ and cloned into the pCS2+ vector. Bold letterings indicate primer overhangs that are also present in the pCS2+ sequence and were used to recombine insert and vector using Gibson assembly (NEB). A similar strategy was used to clone the *myo1D* ORF into pCS2+ using the primers 5′-**GATCCCATCGATTCGA**CAGCTTATTATGGCAGAACACG-3′ and 5′-**AGGCTCGAGAGGCCTT**AAATGGGATCCTGGTCCTCTAG-3′.

To test the potential residual activity of the *Myo1D*^*tj16b*^ mutant protein, the tj16b mutation was introduced into *myo1D*-pCS2+ through amplification with the primers 5′-**GCATAATGTATTTACTAGCC**TTTTTCCAGCTCCACTCTCCC-3′ and 5′-**GGCTAGTAAATACATTATGC**-3′, followed by re-ligation through Gibson assembly (NEB).

### RNA and morpholino injections

*myo1Cb*-pCS2+, *myo1D*-pCS2+, and *myo1D*^*tj16b*^-pCS2+ were linearized with KpnI and RNA in vitro synthesized using the mMessage mMachine SP6 transcription kit (Ambion). The resulting RNAs were injected at 50 ng/µl for *myo1Cb* and 25 ng/µl for *myo1D* and *myo1D*^*tj16b*^. Arl13b-GFP-pCS2+ was linearized with ApaI and transcribed with SP6 RNA polymerase as previously described^28^. RNA was injected at 20 ng/µl. To inhibit *myo1D* function, a translation-blocking morpholino (Gene Tools) with the sequence 5′-ACTTTCGTGTTCTGCCATAATAAGC-3′ was injected at 750 µM. All reagents were injected at the one cell stage at a volume of 0.88 nl together with 0.2% phenol red.

### RNA in situ hybridizations

Whole mount RNA in situ hybridizations were performed according to standard protocols^52^. The following probes were used in this study: *cmlc2*^53^, *foxa1/fkd7*^54^, *lefty1*^55^, *pitx2*^33^, *sox17*^56^, *spaw*^32^, *dand5/charon*^34^. To generate a *myo1D* probe, *myo1D* was amplified from *myo1D*-pCS2+ using the following primers 5′-CGC**TAATACGACTCACTATAGGGAGA**GTTCTAGAGGCTCGAGAGG-3′ and 5′-GGCAAGACCAAAGTTTTCATCCGC-3′. The in situ probe was then synthesized directly from the PCR product using the incorporated T7 promoter (bold lettering). In situ hybridizations were documented using a Leica M205FA stereomicroscope equipped with an Infinity2-5C color CCD (Lumenera).

### Immunohistochemistry

Dechorionated embryos were fixed overnight at 4 °C in PEM (80 mM Sodium-Pipes, 5 mM EGTA, 1 mM MgCl_2_) −4% PFA −0.2% Triton X-100. After washing 2 × 5 min in PEMT (PEM −0.2% TritonX100), 10 min in PEM −50 mM NH4Cl, 2 × 5 min in PEMT and blocking in PEMT −2% BSA, embryos were incubated 2 h at room temperature with primary antibodies. Following incubation, embryos were washed during 5, 10, 15, and 20 min in PEMT, blocked in PEMT −2% BSA, and incubated again with secondary antibodies for 2 h. Embryos were again washed during 5, 10, 15, and 20 min in PEMT, mounted in PEM −0.75% LMP-Agarose and analyzed on a Zeiss LSM710 laser scanning confocal microscope. The following primary antibodies were used in this study: Mouse@ZO-1 (1:250, Invitrogen 33-9100), Mouse@Acetylated Tubulin (1:2000, Sigma T7451), and Rabbit@γ-Tubulin (1:250, Sigma T5192). The secondary antibodies Goat@MouseIgG1-Alexa568, Goat@MouseIgG2b-Alexa647, and Goat@Rabbit-Alexa488 were obtained from Invitrogen and used at a dilution of 1:500.

### Pharmacological treatments

Stock solutions of IBMX (100 mM, Sigma) and Forskolin (10 mM, Sigma) were prepared in DMSO. Ouabain (1 mM, Sigma) was prepared in distilled water. Embryos were treated with 10 µM Forskolin and 40 µM IBMX, or 5 µM Ouabain at bud stage and washed in Danieau medium after imaging at the eight-somite stage.

### Tracking of fluorescent beads in the LRO

Fluoresbrite Polychromatic Red Microspheres 0.5 µm (Polysciences) were manually injected into the KV lumen. The movement of fluorescent beads was imaged on a wide field Zeiss Axio Observer Z1 microscope with a 40x EC Plan-Neofluar PH/DCII NA 0.75 dry objective using an Andor Neo sCMOS camera and a DsRed filter. In this setup we obtained images with a pixel width of 0.1625 µm and recorded the KV flow for 2 min with a frame interval of 0.04 s. In addition, a single brightfield image was acquired to visualize the KV outline. Automated tracking of fluorescent beads was performed using a custom-made ImageJ script (available as Supplementary Software 1 file). The macro generates an automated tracking file that is compatible with the ImageJ MTrackJ plugin. Following automated detection, tracks were manually curated using MTrackJ to correct for any errors of the tracking algorithm and the tracking parameters exported in a .CSV datasheet for further analysis. Using this procedure, an average of 44022 tracking points were obtained per embryo. KV images were oriented precisely along the antero-posterior axis based on the position of the notochord. The center of the KV was determined as the point lying midway between the anterior and the posterior extremity of the KV on a line that extends posteriorly from the anterior pole of the KV. The angular flow velocities displayed in Fig. 4 and Supplementary Figs. 6, 8 were calculated using the center of the KV as the point of origin of a polar coordinate system.

### Quantification of ciliary orientation

KV motile cilia were labeled using Arl13b-GFP RNA microinjection and imaged on a Zeiss 710 laser scanning confocal microscope. The entire KV was scanned with *z*-sections of 0.2 µm distance and a frame interval of 0.48 s. The 488 nm laser line was used to simultaneously record the fluorescent cilia signal as well as a transmitted light image to visualize KV morphology. Movies were then treated in ImageJ with a Mean 3D filter to visualize the ciliary rotation cone and be able to orient it from base to tip (center of the cone). Each cilium was drawn on the KV using the ImageJ arrow tool. Cilia positions and angles were extracted from the arrow ROIs using a custom-made ImageJ script (available as Supplementary Software 1 file). To compare the position and orientation of cilia in different embryos as shown in Fig. 6h–o and Supplementary Fig. 9, the relative positions of different cilia were normalized in the common space of a circular virtual KV. Each cilium was placed on the virtual KV according to its relative radial position with respect to the organ border.

### Quantification of basal body positioning

Confocal *Z*-stacks of the entire KV were acquired with a distance of 0.6 µm between two consecutive planes. The KV was oriented along the antero-posterior axis based on the position of the notochord. Basal bodies were identified as γ-tubulin positive spots that are located at the base of an acetylated tubulin positive ciliary axoneme. The junctional marker ZO-1 was used to outline KV cells and determine the antero-posterior extent of each cell. We restrained our analysis to cells for which the entire outline could be unambiguously identified in the ZO-1 channel. As the KV is an ellipsoid structure, this applies essentially to the cells of the dorsal roof and ventral floor of the KV. Based on this criterium, we were able to determine the antero-posterior position of an average of 19.4 basal bodies per embryo. The antero-posterior location of the basal body was quantified by calculating the ratio between the distance of the basal body from the anterior-most limit of the ZO-1 signal and the full antero-posterior length of a cell inferred from the ZO-1 signal. According to this convention, a value of 0 would indicate a basal body that is located at the anterior extremity of the cell, while a value of 1 would correspond to an extreme posterior location.

### *dand5* and *charon* quantification

*dand5/charon* in situ hybridization pictures were analyzed with ImageJ. A bounding rectangle was fit on the *dand5* expression domain and the mean gray levels of the left and right part of the rectangle measured. *dand5/charon* right/left enrichment was determined as the ratio between right and left mean gray levels as previously described^57^. For the individual display items presented in Fig. 2g–l, n–p, this approach yielded the following values of right/left asymmetry: WT Bud stage (g): 0.9824; WT three somites (h): 1.0295; WT six somites (i): 1.0712; WT eight somites (j): 1.1004; WT ten somites (k): 1.0916 ; WT 14 somites (l): 1.0450; eight-somite stage WT control (n): 1.0896; MZ myo1D (o): 1.0342; MZ myo1D; MZ myo1G (p): 1.0289.

### Quantification of gastrulation stage convergence-extension movements

Bud stage embryos were imaged laterally on a Leica M205FA stereomicroscope and the angle between the anterior extremity of the hatching gland and the posterior extremity of the tail bud was quantified using the ImageJ angle tool. The reference point for the angle measurement was defined by manually fitting an oval ROI to the embryo and using the ImageJ Measure function to determine the coordinates of the centroid.

### Use of research animals

Zebrafish experiments have been performed in accordance with animal welfare guidelines in the iBV zebrafish facility (authorization #A06-088-17) in the context of the authorized animal experimentation project (APAFIS#5521-201605111041958v6), approved by the animal experimentation ethical committee Ciepal Azur.

### Data availability

The authors declare that all data supporting the findings of this study are available within the article and its Supplementary Information files or datasets (for imaging and quantitation that were generated and analyzed during the current study) are available from the corresponding author upon reasonable request.

## Electronic supplementary material

Supplementary Information

Supplementary Software 1

Description of Additional Supplementary Files
