# Supplementary material for: Myosin1D is an evolutionarily conserved regulator of animal left–right asymmetry
Source: Nat Commun. 2018 May 16;9:1942. doi: 10.1038/s41467-018-04284-8 (PMC5955935; doi:10.1038/s41467-018-04284-8)
Supplement: Supplementary file 1 — Supplementary Information [file 41467_2018_4284_MOESM1_ESM.pdf]

Supplementary Information

Myosin1D is an evolutionarily conserved regulator of animal Left-Right asymmetry

Juan T. et al.

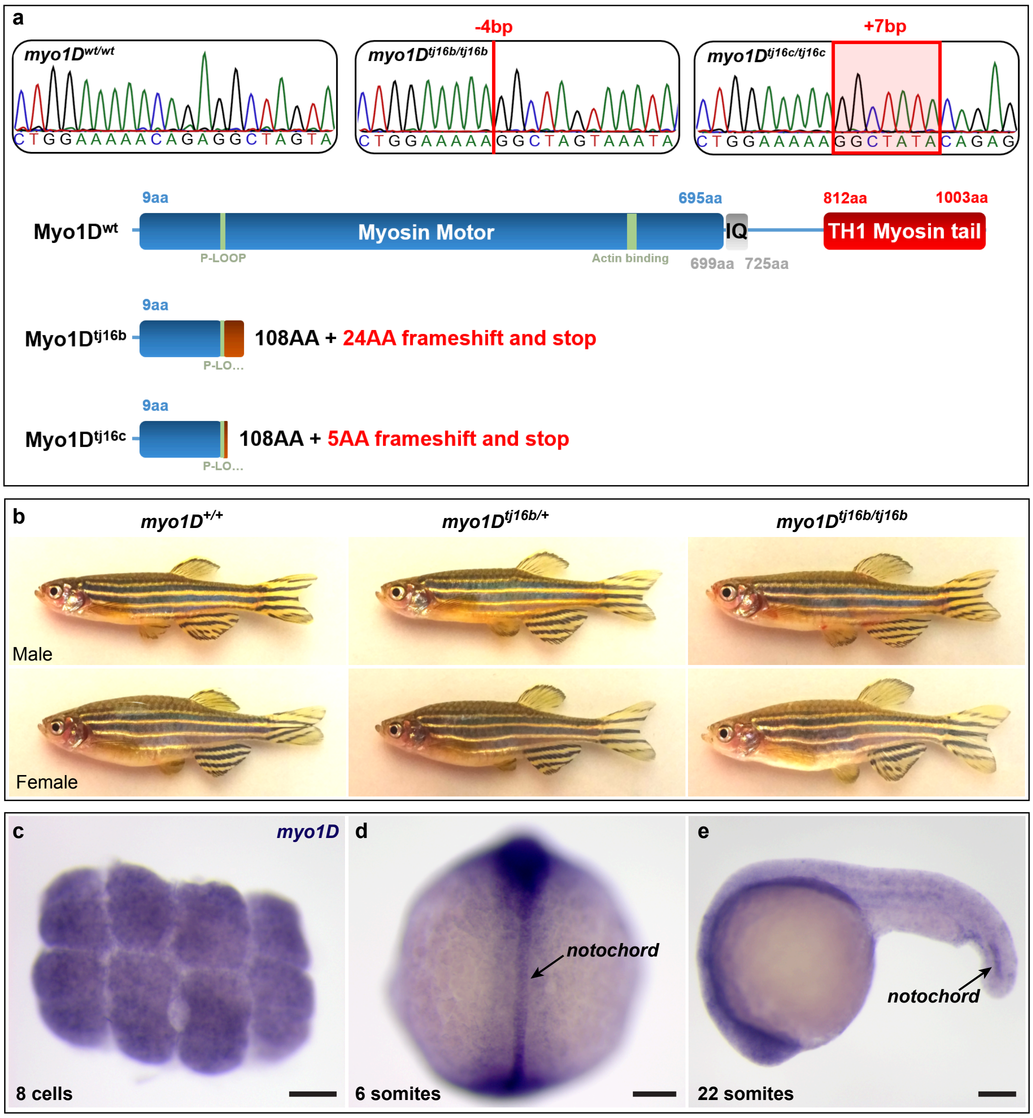

Supplementary Figure 1: Genetic inactivation of zebrafish *myo1D*

a, Sequence chromatograms and schematic representation of *myo1D* mutants generated by Crispr/Cas9 mutagenesis. **b,** *myo1D* homozygous mutant adult fish are indistinguishable from heterozygous or homozygous wild-type siblings. **c,** Whole-mount *in situ* hybridization reveals that *myo1D* transcripts are maternally supplied and can be detected at the 8-cell stage (animal pole view), well before the activation of the zygotic genome. **d, e,** Zygotic *myo1D* transcripts are present at low levels throughout the embryo. At segmentation stages, increased transcript levels are detected in the notochord. **d,** dorsal view, anterior up. **e,** lateral view, anterior to the left. Scale bars: 100 µm.

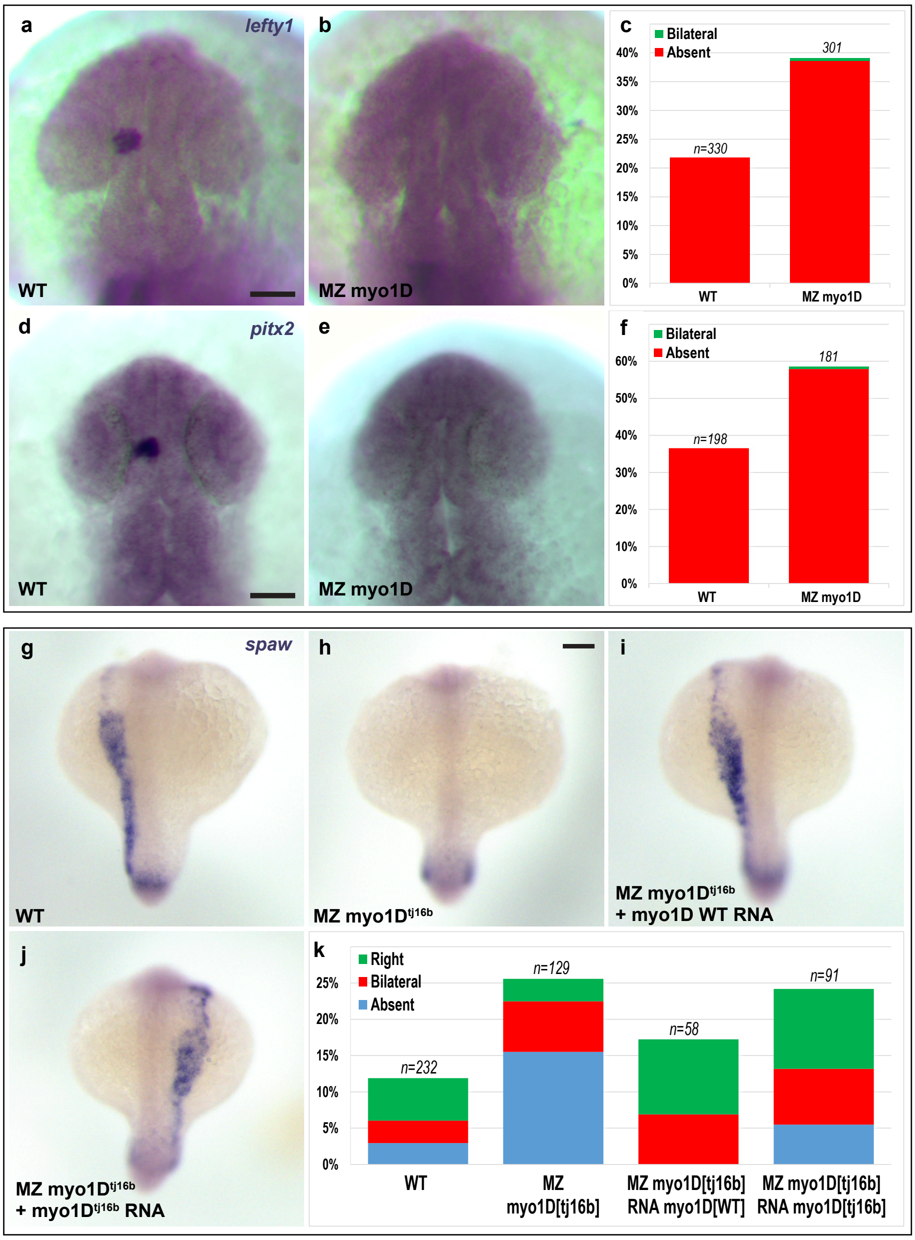

Supplementary Figure 2: Zebrafish *myo1D* controls Left-Right asymmetry in the brain, heart and viscera
a-f, Asymmetric expression of *lefty1* and *pitx2* is impaired in the brain of MZ *myo1D* mutant animals. Dorsal views of *lefty1* (22-somite stage) and *pitx2* (25-somite stage) expression in WT (**a,d**) and MZ *myo1D* mutant (**b,e**) embryos. Quantification of *lefty1* (**c**) and *pitx2* (**f**) expression shows that asymmetric gene expression often fails to be established in mutants. **g-k,** MZ *myo1D* mutants display defects in the asymmetric expression of the nodal-related gene *southpaw (spaw)* in the left lateral plate mesoderm (**h,k**). These defects can be partially rescued by injecting wild-type *myo1D* RNA (**i,k**), but not by *myo1D^{tj16b}* mutant RNA (**j,k**). For the purpose of comparison, we also show the level of *spaw* asymmetry in wild-type embryos, using the dataset also presented in **Fig. 2a-c**. Scale bars: 30 µm in **a,b,d,e**. 50 µm in **g-j.**

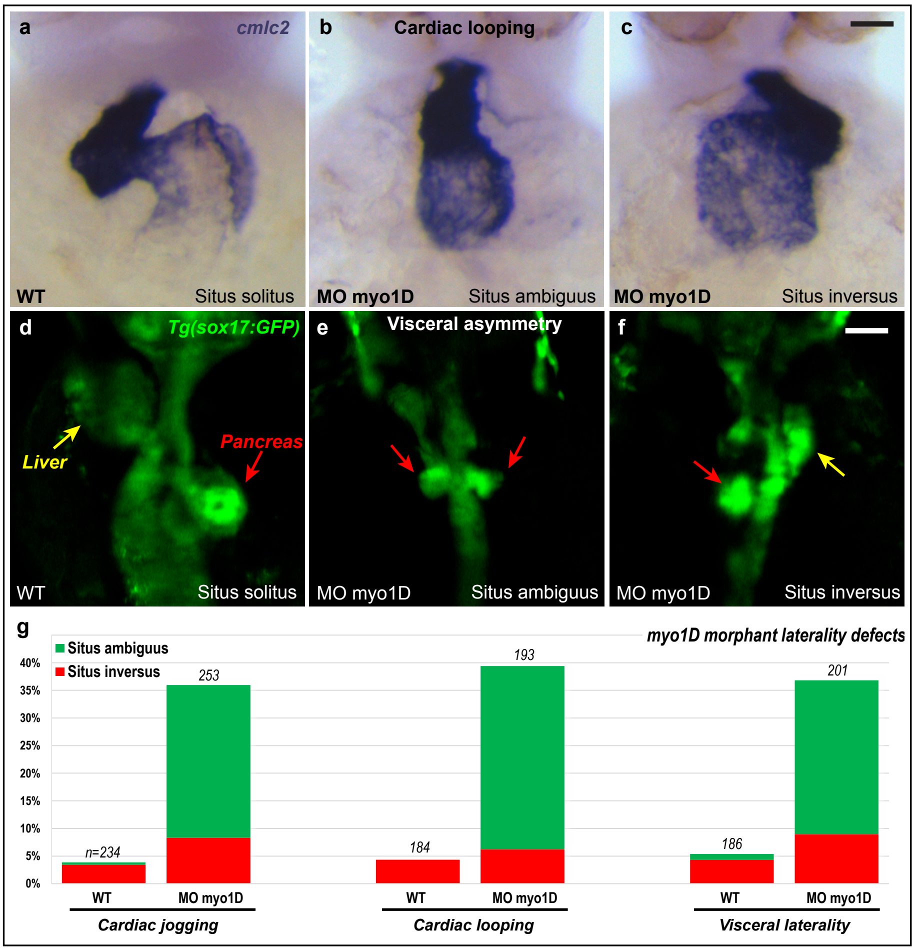

Supplementary Figure 3: *myo1D* morpohlino knock-down elicits Left-Right asymmetry defects
a-g, Morpholino knock-down of *myo1D* elicits laterality defects at the level of the heart and viscera. *myo1D* morphants present defects in cardiac jogging (**g**) and looping (**a-c, g**). **a-c** are frontal views of the *cmlc2*-expressing heart at 48 hpf, dorsal up. **d-f,** *myo1D* knock-down impairs the leftward looping of the gut as well as the lateralized development of the liver (yellow arrows) and pancreas (red arrows). Dorsal views of visceral organs highlighted by an endodermal *sox17:GFP* transgene. Anterior is up. Scale bars: 20 µm in **a-c.** 50 µm in **d-f**.

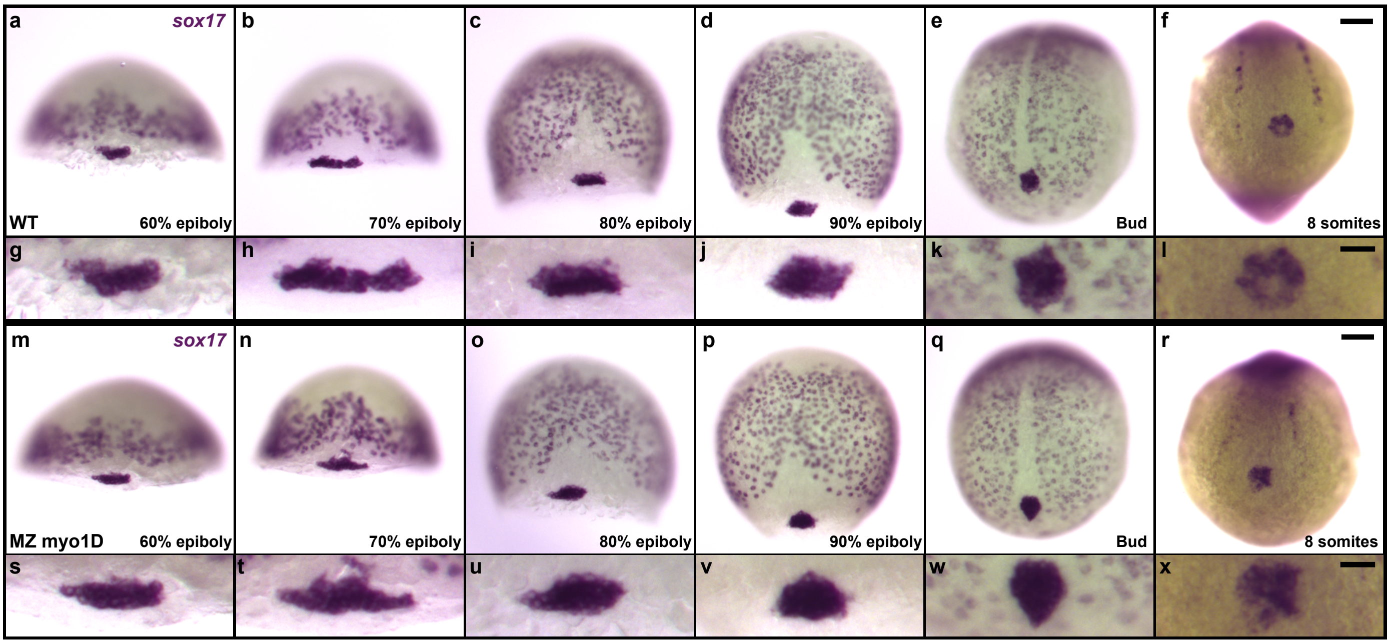

Supplementary Figure 4: Zebrafish *myo1D* is not required for the specification and migration of Left-Right organizer precursor cells
a-x, *sox17 in situ* hybridization marks scattered endodermal cells as well as the posterior cluster of dorsal forerunner cells that give rise to the fish Left/Right Organizer, Kupffer's Vesivle (KV). Comparison of WT (**a-l**) and MZ *myo1D* mutant (**m-x**) embryos reveals no differences in the number and behavior of KV precursor cells. **a-d,m-p,** are dorsal views, anterior up. **e,f,q,r,** are vegetal views of the tail bud region at the end of gastrulation (**e,q**) and the 8-somite stage (**f,r**). Anterior is up. **a-f,m-r** represent low magnification views of the whole embryo. **g-l,s-x** show high magnification views of the KV precursor cells. Scale bars: 50 µm in **a-f,m-r** . 20 µm in **g-l,s-x**.

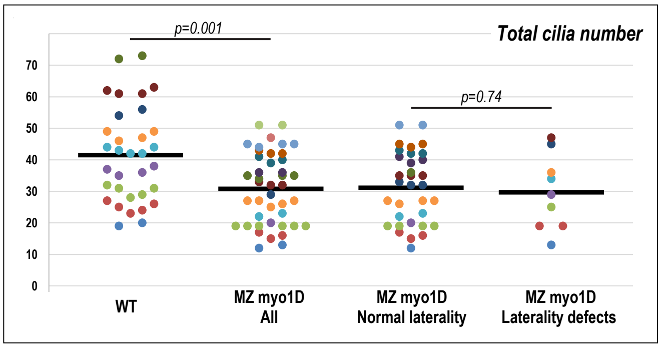

Supplementary Figure 5: Zebrafish *myo1D* controls KV cilia number.
Confocal imaging of *Arl13b-GFP* labelled cilia was used to analyze the number of KV cilia in living embryos. Dot plot representing total cilia number in individual embryos. MZ *myo1D* mutants (n=42) display lower total cilia numbers compared to WT (n=33). Cilia numbers are however similar in MZ *myo1D* mutant embryos with normal or defective laterality. Horizontal bars indicate mean values. All data collected at 8 somites stage. Data were collected from the same embryos that were also used to determine the number of motile cilia displayed in **Fig. 3k**.

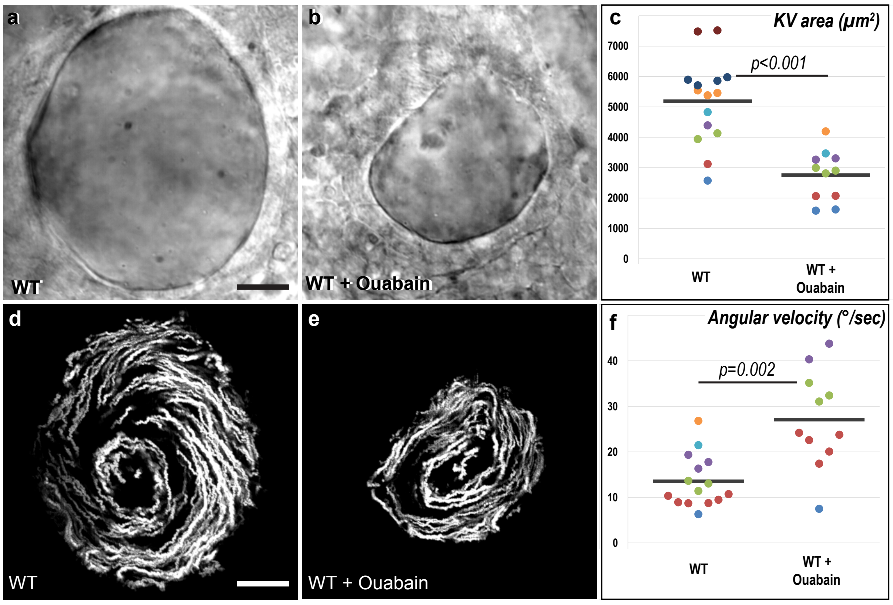

Supplementary Figure 6: The velocity of the zebrafish Left-Right Organizer flow increases upon pharmacological reduction of organ size.

a-c, Ouabain treatment of WT embryos inhibits lumen inflation of Kupffer's Vesicle (KV) and thereby reduces organ size. **a,b,** are brightfield images of the equatorial plane of control (**a,** n=15) and Ouabain-treated embryos (**b,** n=11). **d-f,** Both WT control (**d**) and Ouabain-treated embryos (**e**) display a circular flow pattern. **d,e** are temporal projections of the trajectories of fluorescent microspheres in the KV lumen of the embryos displayed in **a** and **b**. **f,** The angular velocity of the KV flow is increased upon Ouabain treatment. **a,b,d,e** are dorsal views of 8 somites stage KVs, anterior is up. Horizontal grey bars in **c,f** represent mean values. The WT control embryos displayed in this figure are also part of the dataset that is used in **Fig. 4a-h**. Scale bars: 20 µm.

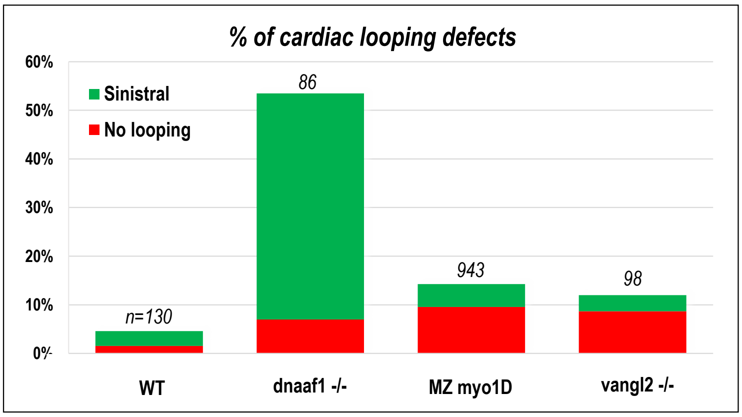

Supplementary Figure 7: Comparative frequency of cardiac looping defects
Comparison of the frequency of cardiac looping defects in WT ABTÜ, as well as *dnaaf1*^{tm317b}, MZ *myo1D*^{tj16b} and *vangl2*^{m209} mutants. The penetrance of cardiac lopping defects is higher in a condition where the KV flow is altogether lost (*dnaaf1*) then when KV flow geometry is impaired (MZ *myo1D*, *vangl2,* **Fig.4d-f**). The MZ *myo1D* mutant dataset is the same that is also displayed in **Fig. 1o**.

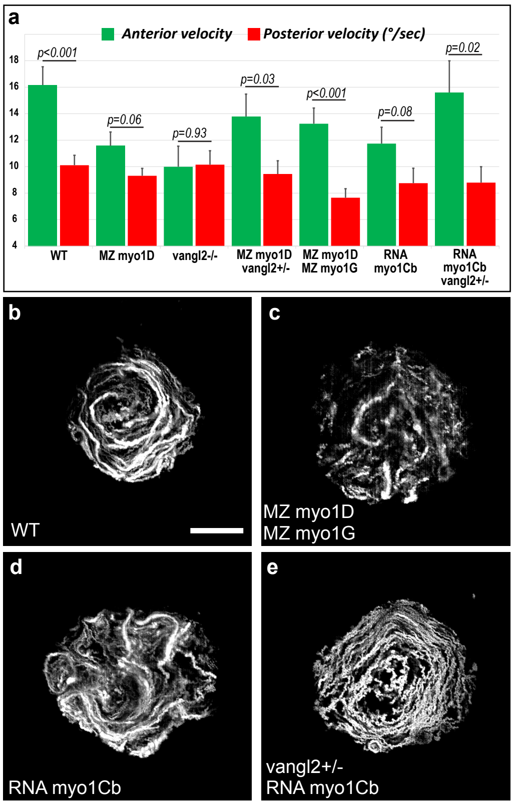

Supplementary Figure 8: *myosin1* and *vangl2* genes control Left-Right Organizer flow
a, WT embryos display a higher flow velocity in the anterior compared to the posterior KV (n=28). This antero-posterior asymmetry is reduced in MZ *myo1D* (n=63) and *vangl2 (*n*=36)* mutants, but restored in MZ *myo1D* ; *vangl2+/- (*n*=25)*. MZ *myo1D* ; MZ *myo1G* double mutants (n=29) maintain a higher flow speed in the anterior KV, suggesting that their LR asymmetry defects are mainly due to the observed overall reduction in KV flow velocity and circularity (**Fig.4h** and **c** below). The antero-posterior velocity gradient is diminished in WT embryos injected with *myo1Cb* RNA (n=37), but restored again upon removal of one copy of *vangl2 (*n*=19)*. **b-e,** Temporal projections of trajectories of fluorescent microspheres in the KV lumen. Compared to WT, MZ *myo1D* ; MZ *myo1G* double mutants or WT embryos injected with *myo1Cb* RNA display altered KV flows. Flow pattern is partially restored when *myo1Cb* RNA is injected in embryos heterozygous for *vangl2*. Dorsal views of 8 somites stage KVs, anterior up. Quantifi-cations and display items are from the same dataset that is also used in **Fig. 4a-h**. Scale bar: 20 µm.

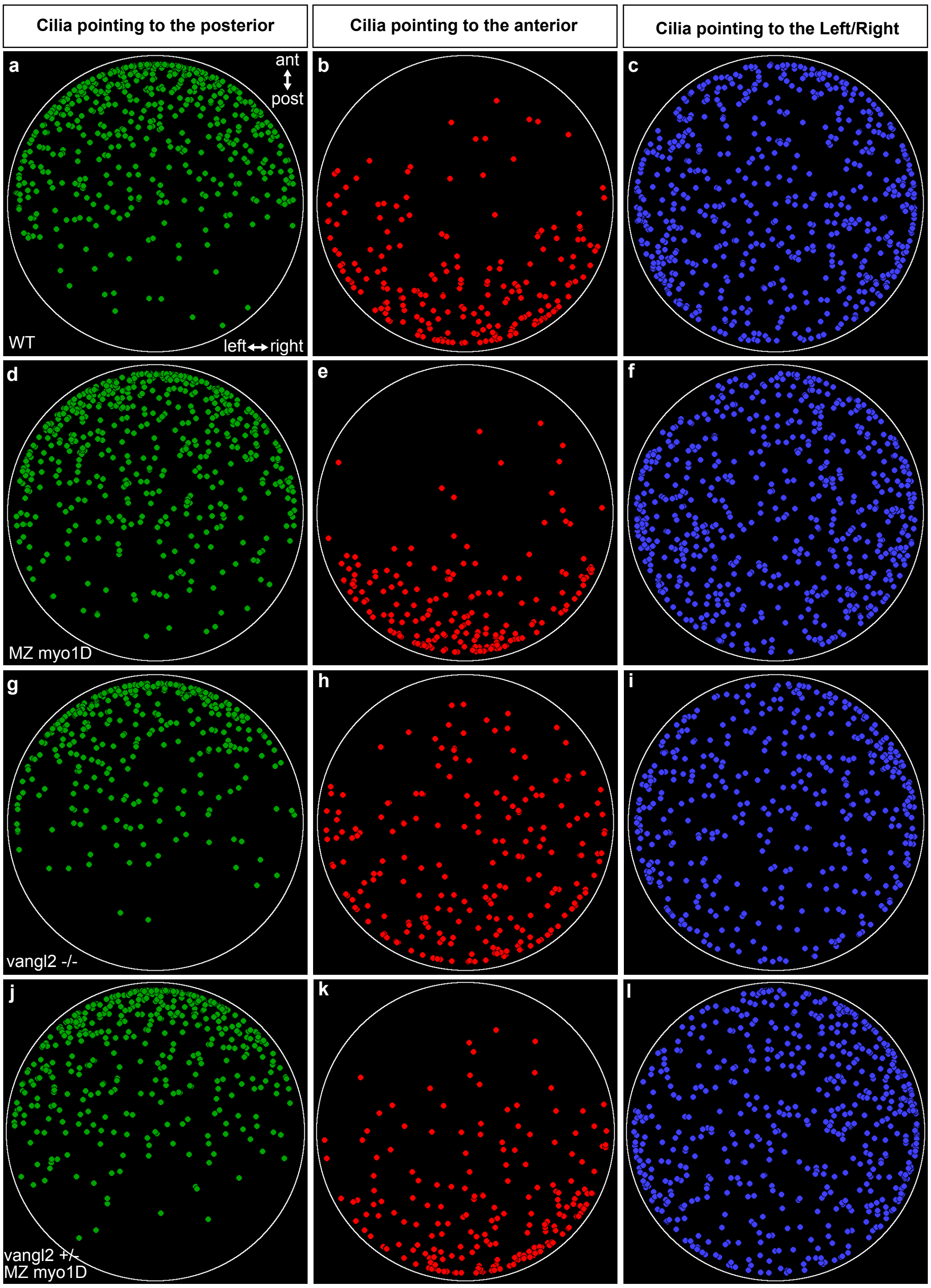

Supplementary Figure 9: Zebrafish *myo1D* and *vangl2* have opposite effects on cilia orientation in the Left/Right Organizer
a-l, The orientation of ciliary rotation cones in Kupffer's Vesicle (KV) was determined using confocal imaging of *Arl13b-GFP* labelled embryos (see Methods). 2-dimensional dot plots indicate the position of cilia within the KV of WT (n=33 embryos/1369 cilia), MZ *myo1D* (n=42/1296), *vangl2-/-* (n=31/966) and MZ *myo1D* ; *vangl2+/-* embryos (n=35/1225). Cilia were categorized according to their orientation: Green dots indicate cilia that point towards the posterior side of the KV with a deviation from the antero-posterior axis of +/-45°. Red cilia point towards the anterior side of the KV with a deviation of +/-45° from the antero-posterior axis. The remaining cilia pointing to the left or right organ wall are blue. **d**, Posteriorly pointing cilia invade the posterior half of the KV in MZ *myo1D* mutants. **h**, An excess of anteriorly pointing cilia is present in the anterior KV of *vangl2* mutants. **j,k,** MZ *myo1D* ; *vangl2+/-* embroys present a WT-like distribution. The plots in this figure correspond to the dataset that is also displayed in **Fig. 6h-o** and are part of the data set used to quantify cilia orientation in **Fig. 6f,g**. To avoid visual crowding in **a-l** and **Fig. 6h-o**, the plots do not represent the complete data set of **Fig. 6f,g**.

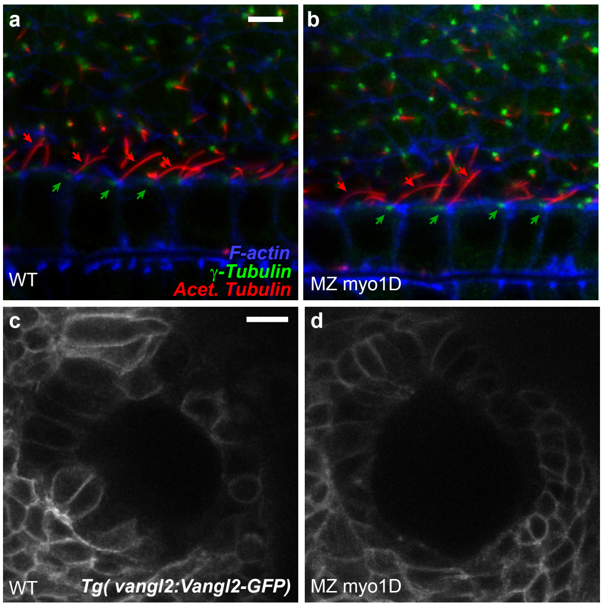

Supplementary Figure 10: Myo1D is not required for floor plate cilia orientation and Vangl2 localisation
a,b, In the floor plate of 22 somite stage WT controls (**a**), 85.3% of the analyzed basal bodies (n=116) are posteriorly localized (green arrows) and 90.6% of the cilia posteriorly tilted (n=117, red arrows). Similarly, 80.5% of the basal bodies (n=164) and 96.8% of the cilia (n=157) are oriented towards the posterior in MZ *myo1D* mutants (**b**). Data were obtained from 18 WT and 22 MZ *myo1D* mutant embryos. Lateral views of the floor plate, anterior to the left. **c,d,** In the Left-Right organizer of both WT (n=15) and MZ *myo1D* mutant (n=20) embryos, Vangl2-GFP displays a similar localisation at the cell cortex. Dorsal views of 8 somites stage KVs, anterior up. Scale bars: 5 µm in **a,b.** 15 µm in **c,d**.

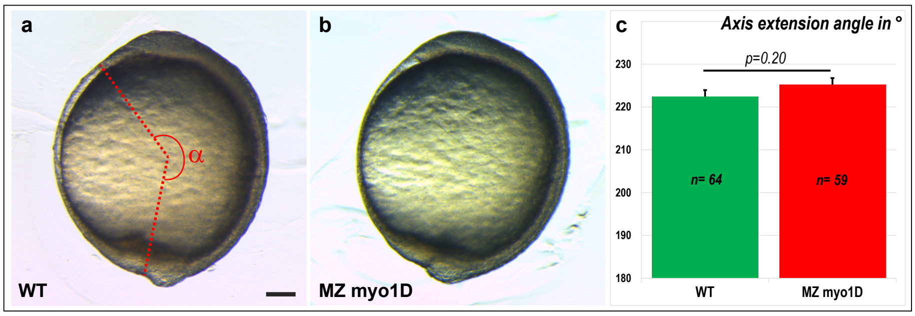

Supplementary Figure 11: Zebrafish *myo1D* is dispensable for gastrulation stage convergence-extension

a-c, *myo1D* is not required for the PCP-dependent convergence-extension movements that drive antero-posterior axial elongation in gastrulation stage zebrafish embryos. Lateral views of bud stage WT (**a**) and MZ *myo1D* mutant (**b**) embryos, anterior up. Axial extension was quantified by measuring the angle between the anterior extremity of the hatching gland and the posterior extremity of the tail bud, as indicated schematically in **a**. **c,** A quantification of bud stage axial extension reveals no significant difference between WT and MZ *myo1D* mutant embryos. Error bars in **c** indicate SEM. Scale bar: 100 µm.
