# Supplementary material for: Myosin1D is an evolutionarily conserved regulator of animal left–right asymmetry
Source: Nat Commun. 2018 May 16;9:1942. doi: 10.1038/s41467-018-04284-8 (PMC5955935; doi:10.1038/s41467-018-04284-8)
Supplement: Supplementary file 2 — Supplementary Software 1 [file 41467_2018_4284_MOESM2_ESM.zip › ImageJ_Scripts/Cilia_Orientation_Read_Me.docx]

**Cilia Orientation – User instructions**

The “Cilia orientation” script allows to analyze the distribution and orientation of cilia in the KV. In a first step, cilia are identified by the user by drawing ROIs for the individual ciliary beating cones. In a second step, the script then performs an automatized analysis of cilia orientation and position.

**I. Input file**

Data have to be provided as a two-channel Z-stacks under TIFF format. The first channel corresponds to the fluorescently labelled cilia, the second channel is a bright-field image of the KV (recorded simultaneously with the fluorescence signal using the transmitted light detector of the confocal microscope). KVs have to be oriented with the anterior pole facing upward in the acquisition files.

**II. Analysis parameters**

The script starts with the definition of the variable “filter_depth”. This parameter defines the Z-radius over which the Mean 3D filter is applied to visualize the ciliary rotation cone. filter_depth is currently set to 5, but depending on the quality of the input file, it may be advantageous to either increase or decrease this parameter for optimal visualization.

**III. Step-by-step procedure to run the script**

1. Open the confocal Z-stack

2. The script now automatically processes the file using a Mean 3D filter to visualize the ciliary beating cones.

3. Select the fluorescent channel and use the slider to move through the Z-stack. Use the ImageJ arrow tool to draw each cilium (from base to tip) and add it to the ROI manager.

4. Once all cilia have been indicated, validate by clicking on “OK”.

5. Select the transmitted light channel, move to the equatorial plane of the KV and click on the anterior pole of the KV. Validate by clicking “OK”.

6. Draw a rectangle bounding the KV and validate by clicking “OK”.

7. The script has now generated three output files:

- A zip folder containing the arrow ROIs of all the individual cilia.

- An Excel file indicating the position and angle of the individual cilia. Angle value range from 0 to 360°, with 0° corresponding to a cilia pointing towards the right KV wall.

- An Excel file indicating the number of cilia pointing anteriorly, posteriorly or in other (lateral) directions, either in the entire KV, or in the anterior or posterior organ half.
