# Supplementary material for: Myosin1D is an evolutionarily conserved regulator of animal left–right asymmetry
Source: Nat Commun. 2018 May 16;9:1942. doi: 10.1038/s41467-018-04284-8 (PMC5955935; doi:10.1038/s41467-018-04284-8)
Supplement: Supplementary file 2 — Supplementary Software 1 [file 41467_2018_4284_MOESM2_ESM.zip › ImageJ_Scripts/KV_Tracker_Read_Me.docx]

**KV tracker – User instructions**

“KV tracker” is a semi-automated script to detect and track fluorescent particles in the KV. While particle detection and track assembly are achieved through a custom-made algorithm, the visualization, editing and analysis of the assembled tracks are achieved using the ImageJ MTrackJ plugin generated by Erij Meijering (<https://imagescience.org/meijering/software/mtrackj/>).

**I. Input files**

The script requires two types of input files:

- First, a TIFF format timelapse movie of the fluorescent beads in the KV (e.g. named XYZ.tif)

- Second a single brightfield image allowing to draw the KV outline (named XYZ_trans.tif)

**II. Analysis parameters**

The script starts with the definition of four parameters that may be adjusted according to the requirements of the analysis:

- The variable “maximal_distance” defines the maximum distance (in µm) that a given particle may travel between two timepoints. If the distance between any two particles at two subsequent timepoints is larger than maximal_distance, the two particles will be considered as belonging to different tracks. In the provided version of the script, maximal_distance is set to 2.2 µm.

- The variable “minimal_frames” defines the minimum number of tracking points for a track. Tracks that contain less then minimal_frames consecutive timepoints are discarded from the analysis. In the provided version of the script minimal_frames is set to 5.

- The variables “particle_size_minimum” (currently set to 10 pixels^2) and “particle_size_maximum” define the size limits for particles that are considered by the automated particle recognition function. Note that particle_size_maximum can be set either to a numerical value, or as is currently the case, to “Infinity”.

**III. Step-by-step procedure to run the script**

1. Open the transmitted light image of the KV (named XYZ_trans.tif)

2. Draw the KV outline and validate by clicking on the “OK” button

3. The script now automatically opens the movie file named XYZ.tif

4. Use the sliders to adjust the threshold values for particle detection, then click on the “Apply” button.

5. Click on the “OK” button of the “Convert Stack to Binary” window

6. Click on the “OK” button to “Adjust threshold for particle selection”

7. The script now performs an automated detection of fluorescent particles and assembles them into coherent tracks.

8. The script then opens the ImageJ MTrackJ plugin interface

9. Click on the “Load” button of the MTrackJ command interface and select the MTrackJ file that has been generated by the tracking script. For a movie named XYZ.tif, this file will be called XYZ_MTrackJ.txt

10. Use the MTrackJ display options to visualize the assembled tracks. If the result is unsatisfactory, start again using by using a different set of threshold parameters for the automated particle detection. Once acceptable parameters have been identified, the MTrackJ “Delete” function can further be used to remove individual erroneous tracks.

11. Click on the “Measure” button of the MTrackJ interface.

12. Click on the “OK” button of “Load track & Measure”

13. In addition to the MTrackJ file, the script has now generated an Excel-readable .csv file (named XYZ_track_points.csv) that contains the spatial and temporal coordinates of all the points of the different tracks as provided by the MTrackJ plugin. This data set can be used by different interfaces (ImageJ, Excel, Matlab…) to quantitatively analyze particle movement in the KV.
