# Supplementary material for: Myosin1D is an evolutionarily conserved regulator of animal left–right asymmetry
Source: Nat Commun. 2018 May 16;9:1942. doi: 10.1038/s41467-018-04284-8 (PMC5955935; doi:10.1038/s41467-018-04284-8)
Supplement: Supplementary file 3 — Description of Additional Supplementary Files [file 41467_2018_4284_MOESM3_ESM.docx]

**Description of Additional Supplementary Files**

File Name: Supplementary Software 1

Description: ImageJ scripts for the analysis of KV fluid flow and cilia orientation. KV tracker: a semi-automated script to detect and track fluorescent particles in the KV. Cilia orientation: a script to analyse the distribution and orientation of cilia in the KV.
